# Twitter Language Use Reflects Psychological Differences between Democrats and Republicans

**DOI:** 10.1371/journal.pone.0137422

**Published:** 2015-09-16

**Authors:** Karolina Sylwester, Matthew Purver

**Affiliations:** Cognitive Science Research Group, Queen Mary University of London, School of Electronic Engineering and Computer Science, Mile End Road, London, United Kingdom; University of Vermont, UNITED STATES

## Abstract

Previous research has shown that political leanings correlate with various psychological factors. While surveys and experiments provide a rich source of information for political psychology, data from social networks can offer more naturalistic and robust material for analysis. This research investigates psychological differences between individuals of different political orientations on a social networking platform, Twitter. Based on previous findings, we hypothesized that the language used by liberals emphasizes their perception of uniqueness, contains more swear words, more anxiety-related words and more feeling-related words than conservatives’ language. Conversely, we predicted that the language of conservatives emphasizes group membership and contains more references to achievement and religion than liberals’ language. We analysed Twitter timelines of 5,373 followers of three Twitter accounts of the American Democratic and 5,386 followers of three accounts of the Republican parties’ Congressional Organizations. The results support most of the predictions and previous findings, confirming that Twitter behaviour offers valid insights to offline behaviour.

## Introduction

Assigning psychological characteristics to political groups is probably as old as politics itself. While in campaigns *ad hominem* remarks about the opponent may not necessarily be supported by evidence, there is a large body of research suggesting that, on average, left- and right-leaning individuals differ in their personalities, how they reason, and how they make decisions. Traditionally, psychological differences between liberals and conservatives have been measured with questionnaires and experiments, methods which may suffer from desirability bias and lack of external validity [1,2]. This study is a linguistic analysis of messages published on the social networking platform, Twitter. We investigate how Democrat and Republican supporters express themselves on Twitter and map the findings to the known psychological differences in political orientation. In the next two sections we summarise the current state of research into the psychology of political orientation and applications of Twitter analyses to psychology research.

### Psychological differences between liberals and conservatives

Traditionally, personality has been measured with the “Big Five” model distinguishing five key personality dimensions: *openness to experience*, *conscientiousness*, *extraversion*, *agreeableness*, and *neuroticism* [3]. Carney et al [4], conducted a multiple study, which showed that *openness to experience* is consistently the best predictor of political ideology, with liberals scoring considerably higher on this dimension. The second most differentiating factor is *conscientiousness*, with conservatives scoring higher than liberals. Other dimensions are much weaker and more inconsistent predictors but liberals tend to score higher on *neuroticism* and lower on *agreeableness*. *Agreeableness*, however, is a multi-faceted factor with components as diverse as altruism and compliance. A study using Italian and Dutch participants found that liberals were more prosocially inclined than conservatives [5]. Further insight comes from a study which investigated various components of *agreeableness* separately, discovering that liberalism was related to compassion whereas conservatism to politeness [6]. A meta-analysis of personality-related findings confirmed that conservatism was negatively correlated with *openness to experience* and risk tolerance, and positively with the need for structure and order [7]. Interestingly, in that paper, the two strongest predictors of conservatism found across multiple studies were death anxiety and system instability.

The Moral Foundations model, developed by Haidt [8], considers psychology differences in the perception of ethical behaviour. Haidt’s model seems to be particularly relevant for investigating political psychology because, although conservatives and liberals may have clashing views on what is or is not moral, each group thinks that their views are just, right and fair. The Moral Foundations Theory identifies six main aspects of morality: *harm*, *fairness*, *liberty*, *ingroup*, *authority*, and *purity*. Liberals score higher than conservatives on the *harm* and *fairness* foundations, but lower on the *ingroup*, *authority*, *purity* and *economic liberty* foundations [9]. Liberals put more emphasis on caring for others and protecting them from harm, as well as executing justice than on the other moral foundations, whereas conservatives are guided by all categories of moral values to a similar extent [10]. In one of their studies, Graham and colleagues analysed transcripts of sermons delivered in liberal (for example Unitarian) and conservative (for example Southern Baptist) churches. The researchers built custom dictionaries reflecting the different moral foundations and used the LIWC software (also employed in this study) to produce word frequencies [11]. They then extracted the most differentiating words with contexts and had three raters assess whether the context was positive or negative. Word frequency analysis yielded support for the direction of differences in *harm*, *fairness*, *authority*, and *purity* but not *ingroup* foundation. Ingroup-related words were used more frequently in liberal than conservative sermons, however, when the context was taken into account it transpired that liberal preachers were rejecting instead of endorsing ingroup values.

Another psychological approach to measuring individual differences is the Basic Personal Values model proposed by Schwartz [12]. The model consists of 10 motivational factors, which account for the wide spectrum of values that drive individual behaviour across cultures. Using a sample of Italian voters Schwartz and colleagues showed that differences in personal values explain a higher proportion of variance in political orientation than the differences in the Big Five. Specifically, left-leaning voters tended to give more importance to *universalism*, *benevolence* and *self-direction*, whereas right-leaning voters put more emphasis on *security*, *tradition*, *conformity* and *achievement* [13]. In line with this finding, in a study where participants were asked to predict political affiliation from photographed faces (which they did with high accuracy) Democrats were perceived as more friendly and Republicans as more powerful [14]. Greater conformity displayed by conservatives corroborates their greater emphasis on group loyalty, as described by Haidt [8]. It is also supported by two other studies showing that liberals perceive themselves as more unique than conservatives [15], and that there is more group consensus among conservatives than liberals [16].

Two other key frameworks in political psychology are Right-wing Authoritarianism (RWA) and Social Dominance Orientation (SDO). RWA focuses on submission to authority, aggression toward out-groups and conventionalism [17]. SDO describes preference for hierarchy and inequality in groups [18]. These two measures are slightly inter-correlated and have been extensively used to explain prejudice. Both RWA and SDO correlate positively with conservative beliefs [18]. Also, both constructs correlate negatively with the Big Five’s *openness to experience*, RWA correlates positively with *conscientiousness* and SDO negatively with *agreeableness* [19]. Interestingly, the usefulness of the Moral Foundations Theory described above has been challenged by the view that liberal-conservative differences in the moral foundations can be explained by differences in RWO and SDO. The high scores in *ingroup*, *authority and purity* foundations were related to higher levels of RWA whereas high scores in *fairness* and *harm* foundations were related to lower levels of SDO [20].

Putting aside the multi-component psychological frameworks, a recent synthesis postulates that the key underlying factor in differences between liberals and conservatives is negativity bias [21]. Higher sensitivity to negative stimuli in conservatives is directly evidenced by studies on disgust using an international sample of respondents from 121 different countries [22]. Hibbing and colleagues also advocate that avoidance of negative stimuli is the reason for conservatives scoring lower on *openness to experience* and higher on *conscientiousness* and conforming to group norms rather than expressing more individualism. Findings suggesting that conservatives are happier than liberals seemingly contradict the negativity bias theory [23,24]. According to Hibbing et al. [21], because liberals expose themselves more often than conservatives to negative stimuli and internalise responses to them, they may be less mentally stable and perceive less life satisfaction than conservatives.

The aforementioned studies heavily rely on the use of questionnaires and, therefore, it is questionable to what extent the elicited responses reflect actual behaviour. The social networking platform Twitter provides a rich source of spontaneous textual data for analysis. The section below describes the ways in which Twitter data has been used in social research.

### Twitter as a source of data about human behaviour

Over the last few years, Twitter has become a prominent data source in the field of sociolinguistics as it captures voluntary opinions and sentiments on a wide range of topics. Information encoded in Twitter data has the potential to unravel the socio-cultural characteristics of users from different areas, for example, the amount of racism and homophobia [25] or may be an accurate surveillance method for mapping the spread of disease [26]. While Twitter users are a self-selected group, there is evidence that analyses of Twitter data produce results congruent with those obtained using standard research methods and data sources [e.g. 27,28].

Twitter provides two types of data for socio-behavioural analysis: non-textual information and the content of tweets. Non-textual information can be derived from a number of features the platform offers. Twitter users can choose to follow other users in order to receive their tweets in a constantly updating feed, the followed users are termed *friends*; they can also themselves have *followers*. An important measure of Twitter activity is the *follower-friend ratio*, that is, how many users follow you (in social network analysis terms, your *in-degree)* in relation to how many users you follow (your *out-degree)*. Users can also create customised reading lists containing selected followed accounts (the purpose might be to group tweets thematically) and subscribe to others’ reading lists. In their tweets, users can *mention* other users by their Twitter username (@username), they can *reply* to others’ tweets and *retweet* others’ tweets; the retweeted tweets will appear in the tweet feed of one’s followers. Twitter messages may contain *hashtags* (#hashtag), user-defined tags categorising the content of the tweet and making it easy to search for tweets referring to the same subject.

One compelling example of using non-textual Twitter data is a cross-cultural comparison of the *pace of life*, *power distance* and *individualism/collectivism* [27]. The researchers found a negative correlation between the temporal unpredictability of tweets and country’s *pace of life* (people from countries with high pace of life tended to tweet at similar times and days); a negative correlation between user mentions and country’s *individualism* (vs. *collectivism*) and a positive correlation between friend-follower ratio (in-degree/out-degree) and the extent to which individuals in a country are comfortable with power imbalance. Another example comes from a cross-cultural study which investigated diurnal and seasonal mood variability using Twitter, corroborating previous results that positive mood is affected by day length and weekday/weekend patterns [28]. Analyses of Twitter usage have also been linked to personality. The number of accounts followed by a user, the number of followers and the number of times a user’s account was listed in others’ reading lists have been found to be accurate predictors of the Big Five traits [29]. The number of followed accounts and the number of followers correlate positively with *extraversion* and negatively with *neuroticism*, influence ratio correlates positively with *conscientiousness*, whereas the number of times an account was listed correlates positively with *openness*.

Socio-psychological as well as commercial analyses of tweet content have predominantly focused on investigating sentiment expressed in tweets. In this type of analysis, words and phrases relating to a given topic are classified as positive, negative or neutral by determining the frequency of different emoticons and/or words with positive and negative valence. A more fine-grained approach is to try to identify complex emotions, topics of interest and attitudes from tweet messages. This can be achieved by determining the frequency of words belonging to different categories for example, religion-related words, government-related words etc. The Linguistic Inquiry and Word Count (LIWC) software enables this kind of analysis by employing a set of dictionaries which group words by category [30]. LIWC can process a text sample outputting frequencies of words from different classes. The language used on Twitter differs from formal written text, often containing misspellings, idiosyncratic vocabulary and linguistic conventions, potentially reducing the accuracy of dictionary-based software like LIWC; however, comparisons against more robust statistical methods suggest that accuracy is very similar when averaged over user profiles [29].

An analysis of tweets with LIWC indicates that they provide cues to self-reported personality traits [31]. *Extraversion* is associated with positive sentiment, religion-related words and assent. *Neuroticism* is associated with 1^st^ person singular pronouns and *openness* is negatively associated with 2^nd^ person pronouns, swear words, affective processes and positive sentiment. A study that inspired this project investigated the happiness of Christians and atheists using their tweets [32]. Christians and atheists were represented by Twitter followers of public figures endorsing Christianity (for example, the pope) and atheism (for example, Richard Dawkins). Using the LIWC software, the study found that Christians were happier than atheists (that is, expressed more positive and fewer negative words in their tweets) and that this difference was driven by their reasoning style. Christians tended to reason more intuitively while atheists were more analytical.

Although discourse analysis is a frequently used method in both political science and psychology, apart from the very recent research on reported vs. expressed happiness [33], no other study has tried to use Twitter to understand personality differences in liberals and conservatives. The social polarisation between Democrats and Republicans has been increasing for the last two decades [34], and is noticeable in other Twitter analyses [35], which suggests these groups are sufficiently distinct to display language differences. In light of the research summarized above, we believe that our analysis provides valuable insights into the psychology of left- and right-leaning individuals.

## Method

### Data collection

The sample consisted of followers of the official Twitter accounts of the Republican and Democratic US Congressional Parties, with the assumption that the majority of Republican followers have conservative views and the majority of Democrat followers have liberal views. It is unavoidable that there will be some noise caused by users following a party whose views they disagree with or by followers with commercial Twitter accounts. However, a similar method of data collection has been previously successfully used to identify Christians and atheists [32], and we validated the data to ensure that followers of each group generally conform to characteristics of Republicans and Democrats (see below).

Using a Python program connected to the Twitter API (https://dev.twitter.com/docs/api), we collected the user IDs of all followers of @GOP, @HouseGOP, @Senate_GOPs (406,687 in total, as of the 9^th^ of June 2014) and @TheDemocrats, @HouseDemocrats, @SenateDems (456,114 in total). Next, we removed the IDs of users following both Republican and Democrat accounts, leaving 316,590 Republican and 363,348 Democrat followers after this filter. We then randomly sampled 17,000 IDs from each follower group and collected timelines and other information about user accounts and tweets. Protected accounts were filtered out, resulting in 13,740 Democrat and 14,363 Republican followers. Due to Twitter API rate limit restrictions, we were able to collect a maximum of 200 tweets for each user. Only the most recent tweets were collected and no content filtering was applied (the analysis was not limited to political tweets). Timeline collection took place between the 15th and 30^th^ of June 2014 and was concurrent with the 2014 World Cup. The influence of this event is particularly noticeable in the tweets of Democrat followers. It is important to note, however, that all tweets were collected over the same period, so differences in the words used reflect different choices, behaviours or interests of users, rather than any difference in availability of events. We applied data cleaning described in S1 Text, which resulted in a dataset consisting of 5,373 timelines of Democrat users with 457,372 tweets in total and 5,386 timelines of Republican users with 466,386 tweets.

### Data validation

A certain amount of noise in the Twitter data is unavoidable but we wanted to ensure that data from the two selected groups of users are comparable and that they conform to expectations based on what we already know about language used by Democrats and Republicans. As a rough validation, we selected words expected to be used more often by one party than the other, based on our own knowledge of issues important to the two political groups and on data reported by www.capitolwords.org about words used by Washington legislators. We then analysed the frequency of use of those buzzwords in our Twitter dataset, which yielded the expected results (see the dictionary in S1 Table for explanation of the terms used). As presented in **Table 1**the odds that users would use the word “benghazi” were 3.93 times higher for Republicans than Democrats, the word “obamacare” 3.36 times higher, and the word “god” 1.40 times higher. Conversely, the odds for the word “birther” were 6.51 times higher for Democrats than Republicans, and the word “bridgegate” 3.70 times higher.

**Table 1:** Fisher's exact tests for political buzzwords, p < 0.001 for all tests.

Buzz word	Count DEM	Count GOP	95% Confidence intervals	Odds ratio
***benghazi***	446	1842	3.544189, 4.370964	3.932325
***obamacare***	868	3068	3.120775, 3.633426	3.365708
***god***	5153	7561	1.348463, 1.447930	1.397302
***birther***	31	5	2.510737, 21.453153	6.512738
***bridgegate***	113	32	2.486100, 5.678139	3.709079

### Analysis

The analysis consisted of three parts: 1) describing the way in which Democrat and Republican users interact on Twitter, 2) investigating the most differentiating words between the two groups, and 3) a timeline content analysis. The third part of the analysis involved finding predictors of political orientation using categories from the Linguistic Inquiry and Word Count (LIWC). LIWC has been validated and successfully used by social science researchers in the past [36]. It contains a set of dictionaries, each describing a different category or words [11]. Some of the categories refer to linguistic concepts (for example, articles), others to various aspects of life (for example, work). LIWC calculates the percentages of words of specified categories appearing in the submitted text. For our analysis, all tweets for each user were concatenated and the resulting timeline was passed for LIWC processing.

### Research hypotheses

Based on the research summarized in the introduction, we developed a number of predictions we tested with the Twitter dataset and LIWC software; these are given in **Table 2**.

**Table 2:** Predictions about language use by liberals and conservatives. The “+” and “-” represent the direction of the expected relationship.

Prediction Category	Measurement Category (with example words)	Prediction
***Uniqueness***	1^st^ person singular pronouns (I, me, mine)	+DEM,-GOP due to higher perception [15] and expression [37,38] of uniqueness in liberals
***Group identity***	1^st^ person plural pronouns (we, our, us)	-DEM, +GOP due to conservatives’ perception of high in-group similarity [15] and consensus [16], and emphasis on in-group loyalty and conformity [10,13]
***Impoliteness***	Swear words dictionary (damn, piss, fuck)	+DEM,-GOP due to reported politeness of conservatives [6]
***Positive sentiment***	Positive emotion dictionary (love, nice, sweet)	+DEM,-GOP due to the finding that liberals express more happiness than conservatives [33], even though the reported happiness of liberals is lower [23,24]
***Negative sentiment***	Negative emotion dictionary (hurt, ugly, nasty)	-DEM, +GOP, due to more frequent negative sentiment expressed in the language of conservatives [33]
***Anxiety***	Anxiety dictionary (worried, fearful, nervous)	+DEM,-GOP due to reported higher neuroticism of liberals [44]
***Feeling***	Feeling dictionary (feels, touch)	+DEM,-GOP due to reported higher compassion and emotionality of liberals [6,45]
***Uncertainty***	Tentative dictionary (maybe, perhaps, guess)	?DEM,? GOP, there is an established relationship between conservative orientation and ambiguity avoidance but it is difficult to predict how it would affect language use [7]
***Certainty***	Certainty dictionary (always, never)	?DEM,? GOP, as above
***Achievement***	Achievement dictionary (earn, hero, win)	-DEM, +GOP due to reported higher emphasis on achievement in conservatives [13]
***Religion***	Religion dictionary (altar, church, mosque)	-DEM, +GOP due to known higher religiosity of conservatives [46]
***Death***	Death dictionary (bury, coffin, kill)	?DEM,? GOP, conservatives report greater death anxiety but it is unclear whether this would lead to more frequent death-related discussions [7]

In some cases, the process of mapping psychological characteristics to language patterns was difficult. One challenge was the ambiguity of findings described in previous research. For example, on the one hand, there are a few studies highlighting liberals’ greater expression [37,38] and perception [15] of uniqueness, while conservatives have a stronger desire for group consensus and sharing the reality with other conservatives [16]. Taken together, these suggest more individualistic talk from liberals and more group-conforming talk from conservatives (see Table 2). On the other hand, research on the “white male effect”, a tendency of white males to be less sensitive to risk than women and minority groups, revealed that this effect is driven by individualistic hierarchists (supposedly a subgroup of conservatively inclined individuals) [39]; this might be taken to suggest more individualistic talk from conservatives. However, the latter study does not directly compare individualistic tendencies between liberals and conservatives, but rather focuses on a subset of conservatives who happen to be individualistic, and it is therefore hard to infer a comparative prediction; we therefore constructed our prediction based on those studies that directly compare the two political groups. The use of the 1^st^ person singular pronoun has been previously linked to gender, age, depression, self-focus and individualism [30,40]; here, we propose the frequency of use of “i”, “me”, “mine” as a predictor of the desire for and expression of uniqueness, a way to emphasise distinctiveness rather than group membership. We interpret the plural counterparts “we”, “us”, “our” as an expression of group identity, as consistently suggested by previous research [30,41–43] (see Table 2).

Another problem we encountered was the difficulty in predicting how some aspects of personality will be reflected in language patterns. Early in our research, we anticipated that conservatives would display more positive sentiment words due to their higher reported happiness [23,24]. However, a recently published study discovered that reported happiness does not translate to expressed happiness, leading us to reverse the direction of our original prediction about positive sentiment [33]. The same study also suggested that conservatives would be more likely to use negative sentiment words, further informing our prediction.

The negativity bias framework proposed by Hibbing et al. [21] did not allow us to make definite predictions. It is unclear whether negativity bias among conservatives will lead to more or less frequent use of negative sentiment words (does higher sensitivity lead to more discussion of negativity, or avoidance thereof?); the same applies to death-related words or words related to certainty and uncertainty. Where possible, we relied on other research to substantiate our predictions [44–46]; for outcomes where we did not find sufficient evidence in the literature, we treated our analysis as exploratory.

## Results

### Characteristics of Twitter user behaviour

**Fig 1:**
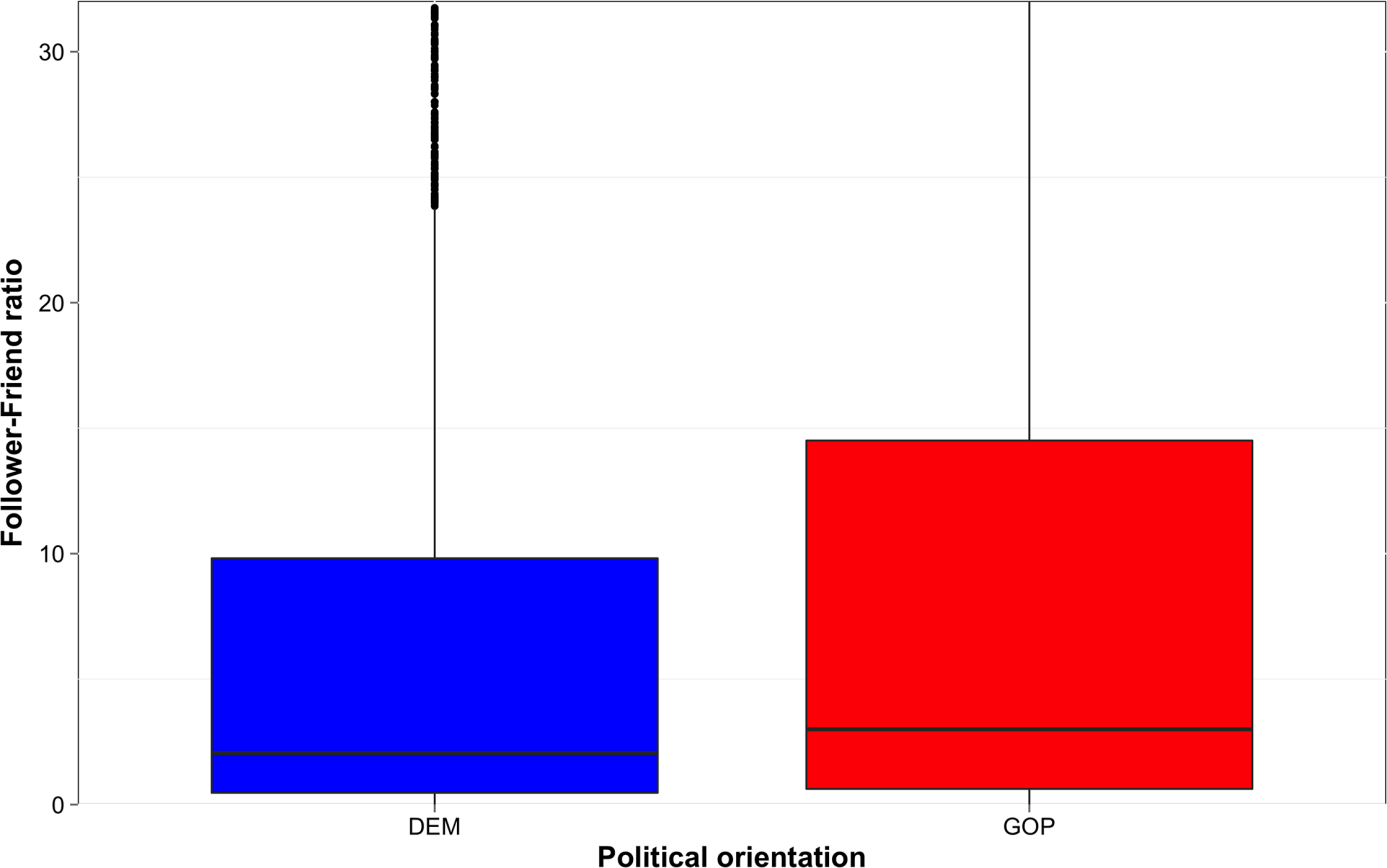
Follower-friend ratio by political orientation. Follower-friend ratio was calculated by dividing each user’s follower count (number of following users) by friend count (number of followed users). Boxplots represent interquartile regions with medians.

We compared follower counts for Democrats and Republicans with a Mann-Whitney U test. On average, Republican users were followed by significantly more accounts than Democrat users (Med_GOP_ = 219, Med_DEM_ = 201, W(10759) = 2618290, Z = 3.4234, p<0.001, d = 0.03), while Democrat users followed significantly more accounts than Republican users (Med_GOP_ = 52, Med_DEM_ = 78, W(10759) = 15583995, Z = 6.9193, p<0.001, d = 0.06). These differences are also visible in Fig 1, which shows ratios obtained by dividing the number of followers by the number of followed accounts.

Followership statistics have been previously discussed by Quercia et al. [29] and have been found to be a good predictor of personality; however, both high follower counts and friend counts were found to predict the same dimensions, correlating positively with *extraversion* and negatively with *neuroticism*, neither of which have been identified as differentiating factors between Republicans and Democrats. Here, we are interested in a possible link with our hypotheses (see Table 2). To explore the relationship with our first two hypotheses concerning self vs. group reference, we therefore correlated the follower-friend ratio with the frequency of using first person singular and plural pronouns. There was a negative relationship between the follower-friend ratio and the frequency of using “i”, “me” and “mine” (r_S_ = -0.33, p<0.001) and a positive relationship with the frequency of using “we”, “us” and “our” (r_S_ = 0.15, p<0.001). This may suggest that users who create or express a sense of group identity by frequent use of 1^st^ person plural pronouns attract larger audiences than those who use 1^st^ person singular pronouns relatively more frequently. Alternatively, users who often say “we”, “us” and “our” may function as group leaders offline and bring a ready-made group of followers to the Twitter network.

**Fig 2:**
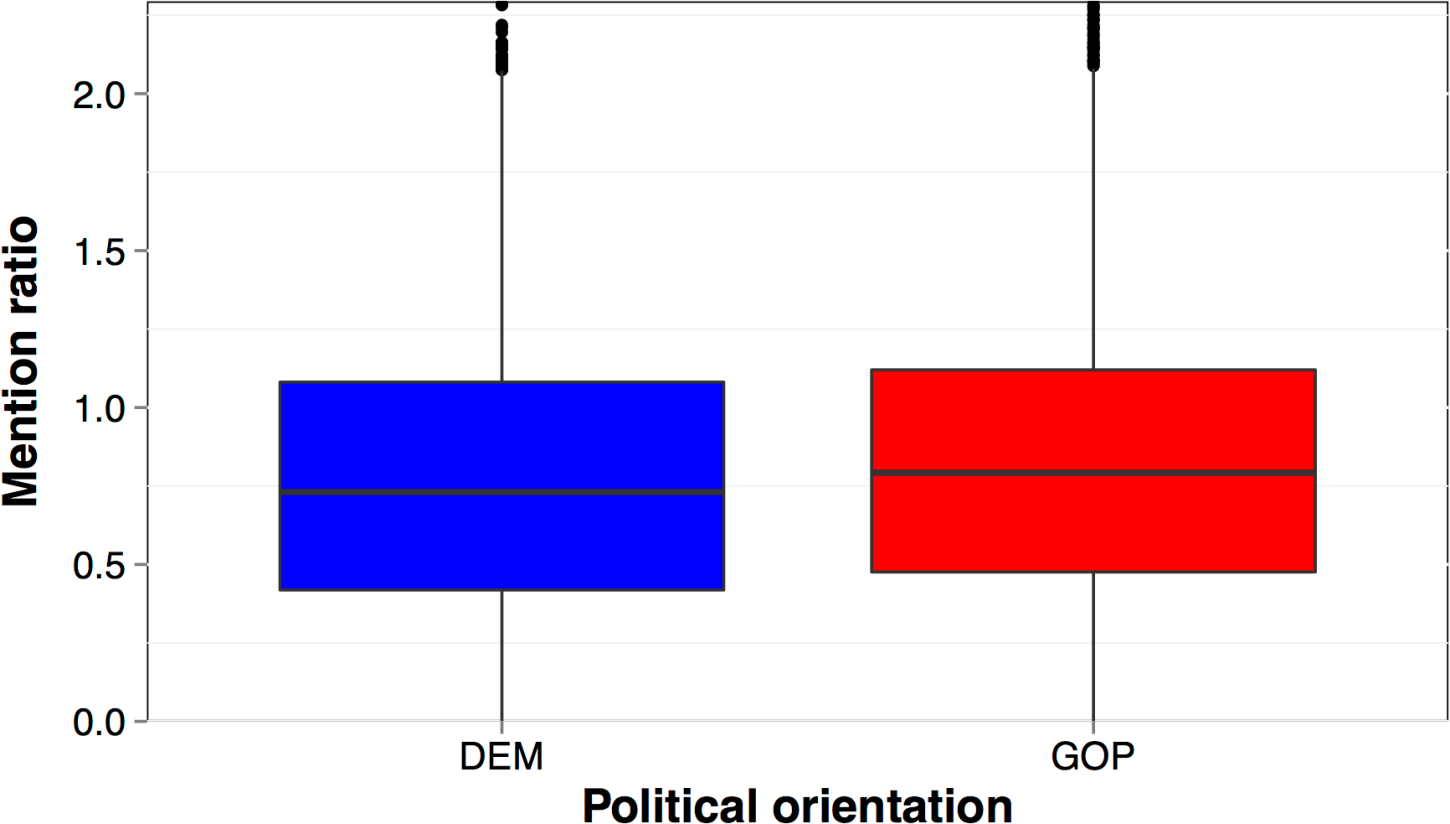
Mention ratio by political orientation. Mention ratio was calculated by dividing the total number of mentions per user by the total number of tweets. Boxplots represent interquartile regions with medians.

Another interesting effect is the difference in the frequency of mentioning other users. The mention ratio was calculated by dividing the total number of mentions (@) by the total number of tweets. On average, Republican users employed mentions significantly more often than Democrat users (Med_GOP_ = 0.79, Med_DEM_ = 0.73, W(10777) = 13738891, Z = 4.82, p<0.001, d = 0.05, Fig 2). While it is tempting to interpret this as relating to higher in-group consensus or collectivism of conservatives [cf. 23], the use of mentions is not in itself related to the use of 1^st^ person plural pronoun (r_S_ = 0.02, p = 0.09); instead we speculate that, taking into account Republicans’ greater emphasis on hierarchy, more frequent use of mentions might reflect their tendency to give credit to or acknowledge others, which may matter in maintaining a more rigid social structure. We also investigated differences between the frequency of linking to websites and re-tweeting messages but did not find significant differences between the two groups.

### Word-frequency analysis

To investigate differences in textual content, we next analysed the most frequently used words, first stemming the words by removing any part of the word other than its root (for example words such as “wait”, “waiting”, and “waited” would all be treated as “wait”). Word stemming is a commonly adopted method in information retrieval because it allows for grouping semantically similar words. The most popular stemming method is Porter’s stemming algorithm, which was employed by the R Snowball C package we used [47]. For comparison, an analysis using unstemmed words can be found in S1 Text. We also removed numeric values and stopwords such as articles and prepositions. Stemming was not applied in the subsequent LIWC analysis.

We employed two methods for finding the top differentiating words for Republicans and Democrats. The first method relies on the difference in proportions. We computed proportions for all word stems with a count of 10 or higher for Republicans and Democrats and subtracted the proportions for one group from the other. We then extracted the 20 words with the highest and lowest difference (**Table 3**). This method can be expressed as the following conditional probability of word use given party affiliation:

$$p(w|pa)=\frac{n(w,pa)}{n(pa)}$$

where n(w|pa) is the count of the number of times a particular word occurs in the tweets of the followers of a given party and n(pa) is the count of all words used in the tweets of followers of that party. Top DEM and GOP words were identified by finding the largest positive and negative difference between p(w|DEM) and p(w|GOP).

**Table 3:** Twenty most differentiating word stems between Democrats and Republicans based on difference in proportions.

Top GOP word	Count GOP	Count DEM	Top DEM word	Count GOP	Count DEM
***obama***	11242	3514	***love***	16778	19732
***tcot***	4099	450	***lol***	6129	8258
***will***	23516	19335	***just***	26654	27678
***god***	7798	5346	***feel***	5386	7109
***obamacar***	3089	879	***fuck***	2183	3852
***america***	3763	1828	***like***	22187	22695
***liber***	2427	621	***realli***	7731	8876
***american***	4383	2732	***watch***	9620	10508
***great***	14825	12711	***n't***	47050	45895
***benghazi***	1845	449	***got***	7805	8578
***tax***	2985	1648	***happi***	7720	8462
***conserv***	1860	627	***shit***	1700	2734
***run***	5288	3940	***worldcup***	1086	2129
***state***	4583	3273	***amaz***	2533	3472
***countri***	3826	2558	***work***	11043	11505
***govern***	2576	1373	***women***	1840	2740
***obam***	1252	280	***day***	17335	17405
***vote***	6348	5148	***know***	14052	14242
***illeg***	1312	379	***much***	7297	7822
***lie***	3027	2009	***life***	6195	6743

The drawback of this method is that it underrepresents the importance of difference in usage with less frequently used words: absolute probabilities will be lower for frequently used words, and thus large differences between them are less likely. The second method, based on weighted frequencies, remedies this problem: the frequency of using each word by each group is divided by the sum of using that word by both groups (**Table 4**). The resulting value is adjusted to account for slightly different sample sizes. Additionally, to account for missing probability mass due to unobserved events, before we conducted the above calculations, we smoothed the data by adding 50 to all counts [48]. The second method can be expressed in terms of the following conditional probabilities of party affiliation given word use:

$$p(pa|w)=\frac{n(w,pa)}{n(w)}$$

where n(w|pa) is the count of the number of times a particular word occurs in the tweets of the followers of a given party and n(w) is the total count for that word used in the tweets of followers of both parties. These proportions were then weighted to account for a small difference in sample size.

**Table 4:** Twenty most differentiating word stems between Republicans and Democrats obtained with 50-smoothing and weighted word frequency method (hashtags excluded).

Top GOP word	Count GOP	Count DEM	Top DEM word	Count GOP	Count DEM
***rino***	339	11	***kenya***	80	315
***bho***	272	14	***tweetdeck***	20	132
***lerner***	326	26	***delhi***	12	105
***clotur***	259	16	***cheney***	99	317
***lib***	708	116	***wat***	57	207
***reid***	720	126	***medit***	48	181
***phoni***	299	33	***smh***	224	591
***defund***	393	61	***favourit***	28	125
***carney***	230	23	***pbo***	13	91
***obamacar***	2089	509	***richi***	21	108
***loi***	238	27	***kenyan***	39	148
***border***	828	191	***arsenal***	53	178
***liber***	2266	586	***album***	330	778
***administr***	867	207	***biafra***	11	82
***pelosi***	274	42	***nene***	18	97
***impeach***	674	162	***realis***	14	87
***psalm***	349	69	***qampa***	18	94
***obama***	10891	3226	***strateg***	62	186
***amnesti***	296	57	***journey***	139	344
***illeg***	1253	369	***maya***	61	181

### Linguistic Inquiry and Word Count (LIWC) analysis

Based on our hypotheses and the results of the word count analysis described in the previous section, a number of LIWC dictionary categories were chosen as predictors of following Democrats or Republicans. Counts of words in these categories were calculated from the unstemmed texts using the LIWC software, and analysed for their predictive association using multiple logistic regression. For all models Republican followers were coded as 0 and Democrat followers as 1 and we adopted a conservative significance level of p<0.01 due to the large sample size. In the initial model with all of the predictors, only some were significant (**Table 5**).

**Table 5:** Initial logistic regression model.

Predictors	Estimate	Standard Error	Z value	P value
***(Intercept)***	0.4711053	0.1192538	-3.95	7.80E-05***
***1st person singular pronouns***	0.1036425	0.0103252	10.038	2.00E-16***
***1st person plural pronouns***	-0.1361112	0.0310361	-4.386	1.16E-05***
***Swear words***	0.2490142	0.0512089	4.863	1.16E-06***
***Positive emotion words***	0.0406131	0.0094521	4.297	1.73E-05***
***Negative emotion words***	-0.0595763	0.0261562	-2.278	0.022744*
***Anxiety words***	0.3952645	0.0916744	4.312	1.62E-05***
***Feeling words***	0.1586861	0.0577905	2.746	0.006035**
***Tentative words***	-0.0908508	0.0272784	-3.33	0.000867***
***Certainty words***	0.0003329	0.0325081	0.01	0.99183
***Achievement words***	0.0250449	0.0226362	1.106	0.26855
***Religion words***	-0.1362726	0.0236901	-5.752	8.80E-09***
***Death words***	0.0887033	0.0815541	1.088	0.276744

Republican followers were coded as 0 and Democrat followers as 1.

Signif. codes: 0 ‘***’ 0.001 ‘**’ 0.01 ‘*’ 0.05 ‘.’ 0.1 ‘ ’ 1

The second model includes only predictors significant at p<0.01 (**Table 6**). A one unit increase in 1^st^ person singular pronouns, Swear words, Positive Emotion words and Anxiety words increases the odds of the user following Democrats by respectively, 11%, 20%, 5% and 35%. A one unit increase in 1^st^ person plural pronouns, Religion words, and Tentative words increases the odds of the user following Republicans by respectively, 14%, 15% and 10%.

**Table 6:** Logistic model including only predictors significant at p<0.01.

Predictors	Estimate	Standard Error	Z value	P value	Odds Ratio
***(Intercept)***	-0.490264	0.092818	-5.282	1.28E-07	0.6124646
***1*** ^***st***^ ***person singular pronouns***	0.102213	0.009959	10.264	2.00E-16	1.1076199
***1*** ^***st***^ ***person plural pronouns***	-0.13309	0.030918	-4.305	1.67E-05	1.1423534
***Swear words***	0.180094	0.04187	4.301	1.70E-05	1.1973295
***Positive emotion words***	0.044791	0.009083	4.932	8.16E-07	1.0458096
***Anxiety words***	0.301711	0.081022	3.724	0.000196	1.3521706
***Feeling words***	0.151838	0.058548	2.593	0.009503	1.1639717
***Tentative words***	-0.09837	0.02689	-3.658	0.000254	1.1033705
***Religion words***	-0.139183	0.023423	-5.942	2.81E-09	1.1493341

The odds ratios were calculated by exponentiating coefficients. Republican followers were coded as 0 and Democrat followers as 1.

**Fig 3:**
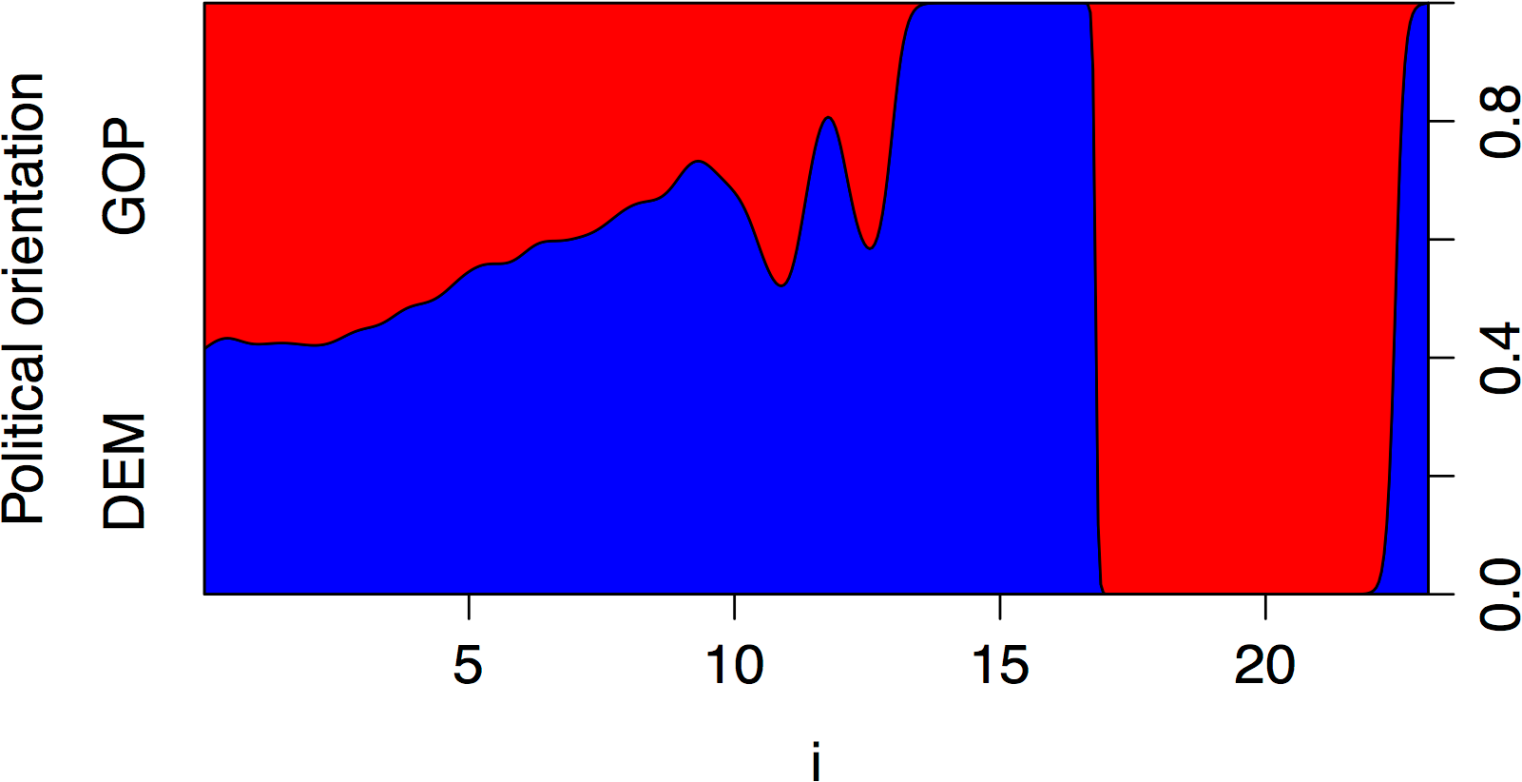
Conditional density plot showing the change in probability of following Republicans vs. Democrats over the frequency of using 1^st^ person singular pronouns. The plot describes how the conditional distribution of political orientation changes over the use of the first person singular pronoun. For example, when the first person singular pronoun is 15, the probability of the political orientation being DEM is 100%, however, this changes as the first person singular pronoun usage increases.

Next, we checked the overall goodness of fit of the model with the le Cessie—van Houwelingen–Copas—Hosmer unweighted sum of squares test [49]. The obtained p value was close to 0, indicating a lack of fit. We visualised the conditional density of the top predictor and found that the relationship was affected by outliers (Fig 3). The probability of following Democrats rather than Republicans increases with the increase in the 1^st^ singular pronoun usage, but at the value of around 17%, the plot flips.

To reduce the expected noisiness of the data, we removed outliers from the next regression. We calculated the interquartile region for each predictor, and excluded any observations with values lower than the 1^st^ quartile–tripled interquartile region and with values higher than the 3^rd^ quartile + tripled interquartile region. This procedure considerably reduced the sample size from10,758 to 4,040 (this is not unreasonable if we assume that each predictor had about 5% of outliers). We reran the original model with the new data (**Table 7**) and excluded predictors that were not significant at p<0.01.

**Table 7:** Logistic regression model with all predictors using data without outliers.

Predictors	Estimate	Standard Error	Z value	P value
***(Intercept)***	-0.32546	0.22806	-1.427	0.153555
***1*** ^***st***^ ***person singular pronouns***	0.08867	0.02054	4.316	1.59E-05***
***1*** ^***st***^ ***person plural pronouns***	-0.3435	0.09793	-3.507	0.000452***
***Swear words***	0.88577	0.21676	4.086	4.38E-05***
***Positive emotion words***	0.13363	0.02517	5.31	1.10E-07***
***Negative emotion words***	-0.12299	0.05362	-2.294	0.021806*
***Anxiety words***	0.85417	0.21939	3.893	9.88E-05***
***Feeling words***	-0.03407	0.14776	-0.231	0.817626
***Tentative words***	-0.11298	0.05405	-2.09	0.036593*
***Certainty words***	-0.03662	0.07287	-0.503	0.615252
***Achievement words***	-0.09091	0.05773	-1.575	0.115288
***Religion words***	-0.22651	0.1444	-1.569	0.116749
***Death words***	-0.28486	0.24512	-1.162	0.245182

Republican followers were coded as 0 and Democrat followers as 1.

Signif. codes: 0 ‘***’ 0.001 ‘**’ 0.01 ‘*’ 0.05 ‘.’ 0.1 ‘ ’ 1

The new model included 1^st^ person singular pronouns, 1^st^ person plural pronouns, Swear words, Positive emotion words and Anxiety words as predictors, however, with the new combination of variables the Anxiety words predictor was only significant at p<0.05, so we also excluded it from the model, resulting in a model displayed in **Table 8**. The sum of squares test gave a p value of 0.52 indicating no lack of fit.

**Table 8:** Final logistic regression model using data without outliers.

Predictors	Estimate	Standard Error	Z value	P value	Odds Ratio
***(Intercept)***	-0.90293	0.14419	-6.262	3.79E-10***	0.4053813
***1*** ^***st***^ ***person singular pronouns***	0.09616	0.01888	5.092	3.53E-07***	1.1009364
***1*** ^***st***^ ***person plural pronouns***	-0.36281	0.09659	-3.756	0.000173***	1.4373656
***Swear words***	0.61113	0.19703	3.102	0.001924**	1.8425114
***Positive emotion words***	0.13338	0.02417	5.518	3.43E-08***	1.1426805

Republican followers were coded as 0 and Democrat followers as 1.

## Discussion

The main goal of this study was to find whether there are differences in language usage between liberals and conservatives expressing themselves on Twitter and whether the direction of these differences matches previous findings in political psychology. Most of our results offer support for the existence of such differences and are in line with the predictions (see **Table 9**).

**Table 9:** Results of the analyses against predictions. Prediction category and outcome columns are in bold if the prediction is supported and not if there is insufficient evidence or if the direction of the prediction was not determined in the first place.

Prediction category (Measurement category)	Prediction outcome	Evidence
***Uniqueness* (1** ^**st**^ **person singular pronouns)**	**+DEM,-GOP**	“I”, “my”, “I’m” and “me” are the most frequently used unstemmed words by Democrats but not Republicans. Frequency of 1^st^ person singular pronoun use is a significant predictor of following Democrats in all regression models.
***Group Identity* (1** ^**st**^ **person plural pronouns)**	**-DEM, +GOP**	“We”, “our” and “us” are among the most frequently used unstemmed words by Republicans but not Democrats. Frequency of 1^st^ person plural pronoun use is a significant predictor of following Republicans in all regression models.
***Impoliteness (*Swear words)**	**+DEM,-GOP**	“Fuck” and “shit” are among the most frequently used stemmed words by Democrats but not Republicans. Frequency of swear words is a significant predictor of following Democrats in all regression models
***Positive sentiment* (Positive emotion words)**	**+DEM,-GOP**	In the most frequently used word stems Republicans use “great” but Democrats use “love”, “like”, “happi” and “amaz”, in unstemmed words Democrats use “lol”. Frequency of positive emotion words is a significant predictor of following Democrats in the regression models including outliers.
*Negative sentiment* (Negative emotion words)	-DEM, +GOP	This prediction is mildly supported: Republicans frequently use “not” (unstemmed words analysis), and often address their adversaries: “obama” “obamacare”, “liberals”, “his”. The first regression shows weakly significant (p<0.05) effect for negative emotion word use predicting Republican affiliation, a consistent, yet not significant trend is present in the model with no outliers.
***Anxiety* (Anxiety words)**	**+DEM,-GOP**	Frequency of anxiety-related words is a significant predictor of following Democrats in three out of four regression models.
***Feeling* (Feeling words)**	**+DEM,-GOP**	“Feel” is one of the top words used by Democrats. Frequency of feeling-related words is a significant predictor of following Democrats in the regression models including outliers.
*Uncertainty* (Tentative words)	?DEM,? GOP	Frequency of tentative words is a significant predictor of following Republicans in the model with outliers.
*Certainty* (Certainty words)	?DEM,? GOP	No effect found.
*Achievement* (Achievement words)	-DEM, +GOP	No effect found.
***Religion* (Religion words)**	**-DEM, +GOP**	“God” and “psalm” are among the top words used by Republicans. Frequency of religion-related words is a predictor of following Republicans in the regression models including outliers.
*Death* (Death words)	?DEM,? GOP	No effect found.

The analysis of the most differentiating words between Democrat and Republican followers (Tables 4 and 5) reflects differences in discussed topics, the importance of various aspects of life, and personality characteristics. In their Twitter messages, Republicans focus on religion (god, psalm), national identity (america, american, liber, countri, border), in-group identity (conserv, tcot—top conservative on Twitter, rino—Republican in name only), government and law (illeg, lie, vote, administr, impeach, defund, clotur) and their opponents (obama, bho, obamacar, reid, pelosi, carney, loi).

Democrats’ most differentiating words are more emotionally expressive (happi, shit, fuck, like, feel, amaz) and reveal their focus on entertainment and culture (worldcup, watch, nene, maya, arsenal, album, journey, tweetdeck, medit) rather than politics, although topics relating to current international affairs are frequently discussed (kenya, delhi, biafra). The word analyses using unstemmed words, described in S1 Text, are broadly in agreement with the stemmed analyses presented above. Table A in S1 Text shows the more common use of 1^st^ person singular pronouns by Democrat followers and 1^st^ person plural pronouns by Republican followers, as well as frequent use of 3^rd^ person masculine pronoun. Surprisingly, the most differentiating word was the “the” article, which qualitative investigation suggests is related to frequent appeal to authority (the lord, the government, the usa, the senate, the law) in conservatives’ messages.

As predicted, the LIWC analysis shows that Democrat followers tend to use 1^st^ person singular pronouns more often than Republican followers, which we interpret as their greater desire for emphasizing uniqueness. Democrats also tend to use words expressing anxiety and feelings. Conversely, the language of Republican followers highlights their group identity, relatively infrequent usage of swear words and religiosity. Our findings corroborate those indicating political differences in the *agreeableness* component of the Big Five, the *in-group* foundation in the Moral Foundations Theory and the *self-directio*n and *conformity* values in the Basic Personal Vales model [4,6,10,13]. These results suggest that language used on Twitter does, indeed, reflect individual differences between liberals and conservatives.

We found that the expression of positive emotions is positively correlated with following Democrats, but not Republicans. This result supports the recent evidence that despite reporting higher life satisfaction (happiness) Republicans express it less (to measure display of happiness the researchers analysed facial expressions, congressional records and tweets [33]). Our result is also in line with the finding that conservatives may, in general, avoid expressing emotions [45]. Research on a sample of Polish students showed that right-wing authoritarianism was negatively associated with positive affect [50]. In another study of autobiographical memories, individuals with more humanist vs. normative ideology reported more joy, distress, fear and shame [51]. The consistency of Democrats using more emotional language in the three LIWC categories: Feeling, Positive Sentiment and Anxiety, leads us to believe that the LIWC swear words category should not be linked to Impoliteness, but rather be considered additional evidence for high emotionality of liberals’ vocabulary. Conservatives evaluate their life satisfaction highly when surveyed: is this an artefact of the self-reporting method used or a true self-perception not captured in language due to its reduced emotional expressiveness? It is also intriguing to imagine what role contextual effects play: had we collected the data shortly after a Republican victory, would we see a different outcome of our sentiment analysis?

For some of the psychological differences we predicted, we found no or a weak effect. It is worth noting that, because of the predominantly survey-based nature of previous research, it may be unrealistic to expect that all predictions will be supported with observational data. Self-reported data suffers from social desirability and recall bias. Even if greater attention to achievement is more frequently reported by Republicans, it may not manifest itself behaviourally. One interesting finding is that, despite the high uncertainty avoidance in conservatives reported in the literature [e.g. 4], Republicans used more tentative words than Democrats. One possible interpretation of this result is that, because of the greater need for ambiguity management and cognitive closure in conservatives, they focus on and discuss events with low predictability [52]. Perhaps conservatives emphasize areas of uncertainty because they perceive them as a threat. In our results, it is also noticeable that Republicans often refer to their adversaries (see **Table 3**), so it may be that uncertainty is expressed in the context of their opponents. Further investigation into this result would require qualitative text analysis.

Using Twitter as our data source has several limitations which might have affected our findings. Firstly, Twitter messages contain noise; some accounts may be run by institutions, not individuals and may contain deliberately designed content. Secondly, Twitter users are a sample that may not be representative of the general population and the topics discussed on Twitter may not be representative of offline conversation topics. According to a report released by an American think tank, the Pew Research Center, only 14% of the adult population in the US uses Twitter and Twitter users are younger, more educated and more affluent than the population average [53]. Thirdly, our analysis relied on simple word count and did not consider the actual meaning of tweets (we excluded all punctuation and emoticons from our analysis). In consequence, we are not able to ascertain whether Twitter users had a favourable or unfavourable opinion about a given topic, not to mention detecting complex content, such as humour or sarcasm. Finally, we collected tweets during a particular period of time and did not examine temporal differences in tweet content. It was clear from the analysis of the most differentiating words that references to both recent political and social events were frequently made. All these limitations may have contributed to small effect sizes we found.

Language encodes who we are, how we think and what we feel. We show that, even in a noisy Twitter dataset, patterns of language use are consistent with findings obtained through classical psychology methods. With social interactions happening online more and more frequently, social networking platforms are becoming another valid dimension for studying human behaviour. As the field wrestles with questions about experimenter degrees of freedom, self-reporting bias, and replication problems, Big Data approaches such as the one employed here have enormous potential to improve the field’s confidence in its findings. Our research also highlights the difficulty of directly translating psychological constructs to language. Does the fact that we did not find strong effects for some of the previously reported differences mean that they might not be real, that they are real but not expressed in language, or that our method did not capture them? In particular, we struggled with the direction of predictions relating to negativity bias, which raises questions about how certain behavioural characteristics are reflected in language.

Our research encourages more investigation into how different social groups express themselves: an interesting extension of this study would be to record how right- and left-leaning proponents speak to see what patterns are present in verbal utterances and how they differ from the patterns found in Twitter messages. Also, by exploring more linguistic categories one might be able to create a more accurate model to predict political orientation. Finally, it would be exciting to investigate how the language of Democrats and Republicans on Twitter changes over time in the context of the 2016 US election. Such research could both enrich current knowledge about the psychology of political ideology and translate into commercial applications.

## Supporting Information

S1 Table Dictionary of terms frequently used by Republican and Democrat followers with example tweets. Most of the definitions rely on Wikipedia.
(DOCX)

S1 Text Data pre-processing and additional analyses. (DOCX)
