# Supplementary material for: Twitter Language Use Reflects Psychological Differences between Democrats and Republicans
Source: PLoS One. 2015 Sep 16;10(9):e0137422. doi: 10.1371/journal.pone.0137422 (PMC4574198; doi:10.1371/journal.pone.0137422)
Supplement: S1 Table — Most of the definitions rely on Wikipedia. (DOCX) [file pone.0137422.s001.docx]

| **Term** | **Definition** |
| --- | --- |
| a2 | Possibly a city in Southeaster Michigan formally known as Ann Arbor. "@MySmiley360 A2: on pizza #PompeianParty" , "headed to A2 Book Festival Saturday June 21", "@MJCaan A2 probably not. #scifichat", "A2 Do mosquitos count? #scifichat" |
| benghazi | In September 2012 the U.S. Diplomatic Mission in Benghazi (Libya) was attacked by Islamist gunmen. Republicans heavily criticized the Obama administration for how the attack was framed and handled. "It's a good news to hear, those responsible for the Americans that were killed in Benghazi are caught. Good luck to them 4d punishment." |
| bho | Barack Hussein Obama, "Just saw Amazon is going out of business. Must be true since every biz that bho visits goes out of biz. Will miss them.", "@rushlimbaugh I am tired of u saying women r dumb enough 2 fall 4 BHO's bull just because we r women. U r so wrong. We r smarter than that." |
| biafra | Radio Biafra (former East Region of Nigeria) broadcasts from London with main objectives to provide a channel for unbiased information and news to all ethnic nationalities living under Nigerian oppression.  “@crystal21210 #biafra is my live  my hope  and without biafra we #indigenouspeopleofbiafra are nothing so there for ,#biafra is were i stand”, “Biafra will be free soon..We are hitting it hard...no man born of a woman can stop us,, @radiobiafralive” “how can you feel when your parents went to the church  and die in bomb blast say no to bokoharam by stand  for #biafra is your freedom” |
| birther | A believer in one or more conspiracy theories, holding that President Barack Obama is not a "natural born" citizen of the United States, and therefore ineligible for the presidency (pejorative). “Why do people actually listen to BUFFOONS LIKE like Trump? More birther nonsense. Really?”.”More embarrassment! RT @thinkprogress: EXCLUSIVE: Oklahoma Congressman comes out as a birther http://t.co/NgxjYqPeju” |
| border | Likely a reference to US-Mexican border and recent immigration crisis/scandal there. “More Americans on unemployment opens up jobs 4 illegal immigrants. WOW! Ms Pelosi, who knew how important keeping the border open could be?”, “#ThisIsNow Shut down the borders by force, #impeach this president for war #crimes as well as violation of the #Constitution and #Scandals” |
| bridgegate | A U.S. political scandal in which a staff member and political appointees of New Jersey Governor Chris Christie (a leading member of the Republican Party) collaborated to create traffic jams in Fort Lee, New Jersey, by closing lanes at the toll plaza to the George Washington Bridge. ”Christie Knew About Bridge Lane Closings, Ex-Port Authority Official Says http://t.co/5tf7dPHP87 #bridgegate #p2b”, “#bridgegate showing more evidence that all Republicans are vindictive assholes.” |
| carney | James "Jay" Carney was the 29th White House Press Secretary. “Listening to jay carney try to whitewash their lying @benghazi”, “Jay carney is such a little weasel. He has to know that every time he steps in front of the mic people laugh at him. #tcot” |
| cheney | Richard Bruce "Dick" Cheney is an American politician and businessman who was the 46th Vice President of the United States from 2001 to 2009, under President George W. Bush. “SMH at dick cheney and his wife for making fools of themselves on none other than #foxnews! Where bigots &amp; racists welcome w/ open arms!!!”, “@mrobtuse I'm tired of cheney &amp; his heart stories. He benefited from good health insurance &amp; scientific research. Is he the only one who can” |
| clotur(e) | Cloture is a motion or process in parliamentary procedure aimed at bringing debate to a quick end. "@SenatorWicker For Heavens sake VOTE NO ON CLOTURE! @SenThadCochran" |
| greta | Greta van Susteren (journalist, host of “On the record”), one of the top 100 most powerful women in the world according to Forbes, criticized a Republican candidate for the U.S. Senate in Texas, Dwayne Stovall. "@realDonaldTrump just heard u on Greta  ur a fucking idiot", "@gretawire Greta don't know why Tommy V said he was in the Sit. Room then switched to \"in the White House\" Watch it again. #raisedeyebrows" |
| irs | Internal Revenue Service, "@townhallcom Reform taxes to a 1-page form, disband the IRS. They overstepped their mission &amp; boundaries. Illegal." |
| knicks | NYC baseball team, “But yea, i was serious when I said I would Shelden Williams back if we get a package deal that Candace Parker plays for the Knicks.” |
| koch | The Koch brothers, billionaire conservative activists. “@edshow By "American people", Boehner really means the Koch Bros.” |
| lerner | Lois Lerner worked for the US Internal Revenue Service, now held in contempt of Congress for refusing to testify at a congressional hearing. She was involved in the IRS 2012 scandal which revealed that some political groups (Republicans among others) were scrutinized more than average when applying for tax exemption. “Mrs lerner is NOT being treated unfairly she waived her right to self incrimination by making factual statements #LoisLernerContempt” |
| lt3, actually: &lt;3 | HTML code for a heart, "i lt3 spock so with all of the prequels this summer are they ever going to do a gone with the wind prequel", "omg lt3 you can always rely on you to say what i'm thinking in 140 sarcastic colorful characters", "@troyesivan Happy Birthday Schnookums!! Love you soo very much! &lt;3 Keep being inspiring and entertaining! xx." "@AngryBlackLady omg, &lt;3 you. Can always rely on you to say what I'm thinking in 140 sarcastic, colorful characters." |
| maya | Maya Angelou was an African-American author, poet, dancer, actress and singer, died in May 2014. "\"If you don't like something change it.If you can't change it change your attitude.\" -Maya Angelou. An exceptional woman that will be missed" |
| morningjo | Morning Joe is a weekday morning talk show on MSNBC, with Joe Scarborough discussing the news of the day in a panel format. "i think the better question is why is anyone calling paulryan articulate morningjoe lastword" |
| msm | Main-Stream Media, non-conservative press organisations, “@RepThomasMassie @MattBevin it really is that simple. Time to get our story out because the msm isn't.” |
| nene | NeNe Leakes, American actress, "NeNe you are a rising star dont get down to the level of some of the housewives. Silence is Golden", "@NeNeLeakes @rcbird1021 NeNe you have always been my favorite keep it classy#bloop" |
| nra | National Rifle Association, primary pro-gun lobby group, “why is it that the nra has banned guns from their annual meetings but they want guns everywhere else", "the nra supports laws to allow hidden loaded guns in bars turning minor scuffles deadly momsdemand indygunsense" |
| padres | American Basesball team, “@berniewilson If u play fantasy baseball... Start whoever is throwing against the Padres that day.” |
| pbo | President Barack Obama."@Kernelwars To many questions and to many interviews. youtube has ALOT on PBO's birth location. Education helps tons dude" |
| pelosi | Nancy Pelosi is a member of the Democratic Party and the highest-ranking female politician in American history. She supports gun control and banning of assault weapons. “@SenatorReid you are a bully and you and pelosi should be fired. You caused all the issues with your hatefulness. Obama stinks.”, “# pelosi we have to pass the bill to see what's in it!! That's reckless irresponsible and that u ignorant pos is what cost us $$$” |
| qampa | Q&A session, &amp; html symbol for ampersand "@danielapmeza I loved you latest Q&amp;A! You're awesome, thanks for sharing so much of you with us! &lt;3 #Pandas", "@beckcomgrp The last few minutes of that Q&amp;A were even more shocking than I would have expected from him." |
| redskins | American football team, “@washingtonpost the redskins should not change their name tell the govt to butt out! Get over it go cry in the corner. #football #realmen” |
| reid | Harry Reid is a Democratic senator from Nevada with strong views about Republicans, for example, he is known for accusing Mitt Romney of not paying taxes. “@Doc_0 harry reid is a liar! bible backs me up on that.” |
| rhoa | TV show: The Real Housewives of Atlanta, "The best part of RHOA reunion? The fact that NeNe is just laaaaaughing her ass off the whole time at these folks" |
| rino | Republican in Name Only, a member of the Republican party who holds leftie views. "@SenatorIsakson @SaxbyChambliss In typical RINO fashion you vote with the stinking Democrats for a crappy budget. You're both frauds" , "@RepPaulRyan I am sad today. I once thought you to be a sound fiscal conservative. I realize now you are just another RINO." |
| romneyryan | A reference to the shared candidacy of Romney and Ryan in the last Presidential election cycle. "@megynkelly My wife and I early voted here in Oregon, Proud to cast our votes for Romney/Ryan","Good morning, patriots! And a fine day it is to restore America. I hear there's a Romney/Ryan revolution underway!" |
| rush | Rush Limbaugh, conservative media star and liberal bogey man. “@SenDonnelly youre on the armed services cmte: plz #StopRush: Remove the Rush Limbaugh Show from taxpayer-funded Armed Forces Network! (posted multiple times by one user).” |
| sec | Possibly, from “secretary”, “@KenWahl1 @libertyladyusa Why scream at congress? the sec of def is responsible to hand out $ accordingly” |
| smh | Acronym for 'shake my head'. “Powder alcohol legal but cannabis still not legal in any form... Really?already being told u can snort it,sneak it in places undetected..smh”, “Only two episode left..smh I wished #TheWalkingDead came on every week...then again, everyday!” |
| tcot | Top Conservative on Twitter, "Has anyone else noticed there were no bed bug infestations prior to the \"election\" of Barack HUSSEIN Obama? Just sayin'. *TCOT #TEAPARTY", "O sign off 2 grab Benghazi killer is nt a brave action 2 B applauded! Fox interviewd guy a wk aftr! It's bn 2 yrs! Credit 2 Military tcot" |
| tweetdeck | TweetDeck is a social media dashboard. White House TweetDeck account was hacked in June 2014, might be due to a user who posted multiple messages similar to this one: "Hate Lauren  #OppositeWorlds Fighting fire with Fire. Use TweetDeck HATE Lauren ~'1" |
| va | Veterans Administration, “Failure of the primary mission at the VA – vets died while #VA bureaucrats obsessed over #green #energy - http://t.co/fPzTXQkeg4 #TimCook” |
