# Supplementary material for: Twitter Language Use Reflects Psychological Differences between Democrats and Republicans
Source: PLoS One. 2015 Sep 16;10(9):e0137422. doi: 10.1371/journal.pone.0137422 (PMC4574198; doi:10.1371/journal.pone.0137422)
Supplement: S1 Text — (DOCX) [file pone.0137422.s002.docx]

## Data pre-processing

The collected data was stored in a MySQL database and selected columns listed in Table A were imported from there to an R environment for analysis. Before processing, the dataset contained 1,709,101 tweets from Democrat followers and 1,758,111 from Republican followers. We conducted the following procedures to clean the data.

1. Use only printable text, i.e. letters, numbers and punctuation (this procedure cleans the tweets of emoticons that use uncommon characters. Although emoticons are important part of sentiment analysis, they are not analysed in this project because the focus is on content, not affect).
2. Select users who indicated English as their language (this point as well as 3, 6, 8, 9, and 10 are following Ritter, Preston, & Hernandez, 2013).
3. Choose users with at least 25 tweets in their timeline (to ensure a sufficiently text sample from each user).
4. Choose users whose most recent tweet was not older than the 1^st^ of April 2014 (to ensure that results are comparable).
5. Remove retweets. (Althought the patterns of using retweets may be interesting in their own right, for example, how similar is the language of retweets to original user tweets, for this project including retweets would introduce additional confounding factors. If language patterns can tell us something about a user’s personality, the fact that a user agrees with and retweets a message does not mean that she/he would express the content of the retweet it in the same way. Analysing retweets would mean adding language patterns of other users to a users’ timeline).
6. Remove tweets containing URLs. These tweets will often contain text produced not by the user but by the website to which URL links (a separate analysis of URLs might provide some interesting information about the websites Democrats and Republicans visit).
7. Choose tweets classified as English by the default Twitter language identifier or by an alternative identifier, the Compact Language Detector library in Python.
8. Remove usernames mentioned in tweets (any words or numbers preceded by “@”; this technique will also remove some non-usernames, for example “@7pm”).
9. Remove punctuation and white space, but leave apostrophes and intra-word hyphenation.
10. Remove multiple and trailing spaces and convert all letters to lowercase.
11. Remove hashtags for some word frequency analyses. Hashtags may appear as functional words within a sentence or outside of a sentence, acting as a marker of a specific topic, in which case they may be acronyms or neologisms. Analyses below document whether or not hashtags were removed.
12. Re-check if there are still at least 25 tweets per user for analysis.

After data cleaning, the dataset consisted of 5,373 timelines of Democrat users with 457,372 tweets in total and 5,386 timelines of Republican users with 466,386 tweets.

Table A Variables for analysis obtained with the Twitter API

| **Variable** | **Description** |
| --- | --- |
| tweet_id | A unique identifier for each tweet (numeric) |
| text | Tweet message (character) |
| source | A device from which the message was posted (character) |
| in_reply_to_user_id | Whether the posted message was a reply (boolean) |
| user_id | A unique identifier for each user (numeric) |
| tweet_created | Date when the tweet was created (character) |
| lang | Language of tweets determined by Twitter API (character) |
| cld_lang | Language of tweets determined by Compact Language Detector (character) |
| name | User name (character) |
| location | Location associated with user account provided by the user (character) |
| user_lang | Language detected by Twitter API (character) |
| statuses_count | Number of tweets in a user’s timeline (numeric) |
| followers_count | Number of users’ followers (numeric) |
| friends_count | Number of accounts followed by a user (numeric) |
| account_created | Date of account creation (character) |
| followerOf | Political account (DEM or GOP) that a user follows (character) |

**Analysis with unstemmed words**

The most frequently used category of words are pronouns, which fill spaces between content words. Pronouns make a large part of our unstemmed word corpus and although seemingly lacking content, they contain a surprising amount of information (for example, they can reveal gender, personality and truthfulness, Pennebaker, 2011). We produced two frequency tables (Table B and Table C) using the methods described in the main body of the paper with raw corpus (unstemmed words).

Table B Twenty most differentiating unstemmed words between Democrats and Republicans based on difference in proportions

| **Top GOP word** | **Count GOP** | **Count DEM** | **Top DEM word** | **Count GOP** | **Count DEM** |
| --- | --- | --- | --- | --- | --- |
| ***the*** | 218585 | 197455 | ***i*** | 125989 | 141147 |
| ***obama*** | 11001 | 3324 | ***my*** | 42655 | 49963 |
| ***he*** | 20672 | 14901 | ***i'm*** | 18136 | 22557 |
| ***we*** | 29193 | 24177 | ***me*** | 25792 | 29336 |
| ***tcot*** | 4098 | 450 | ***so*** | 26433 | 29656 |
| ***our*** | 13433 | 9399 | ***love*** | 13663 | 16244 |
| ***will*** | 23060 | 18986 | ***but*** | 23157 | 24522 |
| ***his*** | 12428 | 9497 | ***lol*** | 6126 | 8250 |
| ***us*** | 10748 | 8001 | ***just*** | 26647 | 27673 |
| ***is*** | 90816 | 83880 | ***t's*** | 30040 | 30790 |
| ***not*** | 34252 | 30311 | ***like*** | 20818 | 21408 |
| ***obamacare*** | 3062 | 865 | ***really*** | 7728 | 8875 |
| ***god*** | 7554 | 5146 | ***do*** | 36202 | 35771 |
| ***of*** | 84122 | 77759 | ***it*** | 55037 | 53584 |
| ***for*** | 73157 | 67498 | ***u*** | 10449 | 11202 |
| ***america*** | 3713 | 1783 | ***n't*** | 47050 | 45895 |
| ***irs*** | 1820 | 219 | ***yo*** | 8900 | 9677 |
| ***in*** | 79398 | 73808 | ***this*** | 35732 | 35107 |
| ***if*** | 21934 | 19332 | ***feel*** | 3295 | 4345 |
| ***has*** | 14227 | 12077 | ***got*** | 7788 | 8547 |

Table C Twenty most differentiating unstemmed words between Republicans and Democrats obtained with 50 smoothing and weighted word frequency method (hashtags excluded)

| **Top GOP word** | **Count GOP** | **Count DEM** | **Top DEM word** | **Count GOP** | **Count DEM** |
| --- | --- | --- | --- | --- | --- |
| ***irs*** | 1248 | 183 | ***wat*** | 46 | 165 |
| ***libs*** | 454 | 44 | ***captivity*** | 13 | 91 |
| ***bho*** | 254 | 14 | ***album*** | 293 | 715 |
| ***cloture*** | 259 | 16 | ***favourite*** | 22 | 108 |
| ***liberals*** | 853 | 158 | ***a2*** | 15 | 92 |
| ***illegals*** | 347 | 43 | ***qampa*** | 16 | 94 |
| ***reid*** | 665 | 119 | ***2003*** | 35 | 134 |
| ***phony*** | 285 | 30 | ***biafra*** | 11 | 82 |
| ***administration*** | 766 | 154 | ***kenya*** | 76 | 299 |
| ***obama's*** | 1102 | 240 | ***tweetdeck*** | 20 | 129 |
| ***border*** | 646 | 127 | ***civility*** | 25 | 139 |
| ***lerner*** | 226 | 21 | ***cheney*** | 84 | 276 |
| ***obamacare*** | 2067 | 500 | ***meditation*** | 40 | 162 |
| ***wh*** | 602 | 121 | ***smh*** | 224 | 589 |
| ***psalm*** | 281 | 38 | ***arsenal*** | 48 | 178 |
| ***defund*** | 322 | 49 | ***strategic*** | 47 | 174 |
| ***lois*** | 235 | 26 | ***lt3*** | 462 | 1132 |
| ***proverbs*** | 357 | 63 | ***pbo*** | 12 | 83 |
| ***carney*** | 206 | 22 | ***nene*** | 17 | 93 |
| ***pelosi*** | 261 | 40 | ***dam*** | 34 | 129 |

A surprising finding in Table B is that the article “the”, the most frequent word in English, seems to be used much more frequently by Republican than Democrat followers. To investigate this further we selected all bigrams in which “the” was the first word and produced bigram frequencies. From Table D and Table E, it is clear that Republican followers tend to discuss topics related to politics much more frequently than Democrat followers. The definite article is used when the noun following it refers to a specific well-defined entity (for example, “the lord”, “the usa”, “the government”, “the constitution”). Familiarity and uniqueness are thought to be the main conditions for the definite article usage (Birner & Ward, 2012). A possible interpretation of the differences may relate to relatively low *openness to experience* of conservatives. If Republican followers tend to refer to familiar, known concepts more often than Democrat followers, this may result in differences in the usage of “the”. It is also noticeable that Republicans’ Twitter language and the topics they discuss are much more formal than Democrats’ – frequent use of the definite article might refer to appeal to authority in conservative arguments.

Table D The most differentiating bigrams beginning with “the” between Democrats and Republicans based on difference in proportions

| **Top GOP bigram** | **Count GOP** | **Count DEM** | **Top DEM bigram** | **Count GOP** | **Count DEM** |
| --- | --- | --- | --- | --- | --- |
| ***the irs*** | 611 | 79 | ***the best*** | 3451 | 3798 |
| ***the lord*** | 894 | 370 | ***the gop*** | 722 | 1101 |
| ***the constitution*** | 577 | 138 | ***the world*** | 2599 | 2709 |
| ***the left*** | 524 | 132 | ***the y're*** | 1520 | 1695 |
| ***the truth*** | 1085 | 642 | ***the most*** | 1497 | 1626 |
| ***the people*** | 1620 | 1127 | ***the fuck*** | 216 | 427 |
| ***the obama*** | 386 | 50 | ***the new*** | 1585 | 1655 |
| ***the american*** | 723 | 365 | ***the first*** | 1935 | 1923 |
| ***the follow*** | 1440 | 1056 | ***the same*** | 2754 | 2654 |
| ***the government*** | 594 | 306 | ***the end*** | 864 | 942 |
| ***the dems*** | 327 | 82 | ***the worst*** | 726 | 813 |
| ***the usa*** | 567 | 319 | ***the show*** | 662 | 743 |
| ***the white*** | 486 | 251 | ***the last*** | 1411 | 1417 |
| ***the border*** | 249 | 43 | ***the fact*** | 508 | 599 |
| ***the wh*** | 240 | 40 | ***the time*** | 1063 | 1093 |
| ***the law*** | 531 | 325 | ***the r's*** | 542 | 611 |
| ***the state*** | 587 | 382 | ***the poor*** | 196 | 284 |
| ***the va*** | 262 | 89 | ***the day*** | 1433 | 1401 |
| ***the senate*** | 458 | 275 | ***the shit*** | 98 | 191 |
| ***the democrats*** | 236 | 79 | ***the whole*** | 635 | 671 |

Table E The most differentiating bigrams beginning with “the” between Republicans and Democrats obtained with the previously used 50 smoothing and weighted word frequency method

| **Top GOP bigram** | **Count GOP** | **Count DEM** | ***Top DEM bigram*** | **Count GOP** | **Count DEM** |
| --- | --- | --- | --- | --- | --- |
| ***the irs*** | 611 | 79 | ***the nats*** | 24 | 92 |
| ***the obama*** | 386 | 50 | ***the padres*** | 18 | 79 |
| ***the constitution*** | 577 | 138 | ***the fuck*** | 216 | 427 |
| ***the wh*** | 240 | 40 | ***the rush*** | 12 | 58 |
| ***the border*** | 249 | 43 | ***the uk*** | 67 | 145 |
| ***the left*** | 524 | 132 | ***the album*** | 16 | 59 |
| ***the dems*** | 327 | 82 | ***the shit*** | 98 | 191 |
| ***the five*** | 175 | 30 | ***the koch*** | 50 | 109 |
| ***the msm*** | 114 | 13 | ***the knicks*** | 50 | 107 |
| ***the redskins*** | 148 | 38 | ***the wealthy*** | 19 | 58 |
| ***the lord*** | 894 | 370 | ***the science*** | 27 | 69 |
| ***the va*** | 262 | 89 | ***the nra*** | 55 | 112 |
| ***the democrats*** | 236 | 79 | ***the library*** | 94 | 168 |
| ***the liberal*** | 139 | 39 | ***the gop*** | 722 | 1101 |
| ***the liberals*** | 80 | 15 | ***the movement*** | 16 | 48 |
| ***the obamacare*** | 96 | 24 | ***the iraq*** | 35 | 76 |
| ***the vp*** | 88 | 20 | ***the following*** | 44 | 87 |
| ***the democrat*** | 75 | 15 | ***the universe*** | 84 | 144 |
| ***the sec*** | 135 | 49 | ***the music*** | 113 | 185 |
| ***the american*** | 723 | 365 | ***the journey*** | 26 | 59 |

**References**

Birner, B., & Ward, G. (2012). Uniqueness, familiarity, and the definite article in English. In *Proceedings of the Annual Meeting of the Berkeley Linguistics Society* (Vol. 20).

Pennebaker, J. W. (2011). *The secret life of pronouns: what our words say about us* (1st U.S. ed). New York: Bloomsbury Press.

Ritter, R. S., Preston, J. L., & Hernandez, I. (2013). Happy Tweets: Christians Are Happier, More Socially Connected, and Less Analytical Than Atheists on Twitter. *Social Psychological and Personality Science*. http://doi.org/10.1177/1948550613492345
